# Deep learning approach to detection of colonoscopic information from unstructured reports

**DOI:** 10.1186/s12911-023-02121-7

**Published:** 2023-02-07

**Authors:** Donghyeong Seong, Yoon Ho Choi, Soo-Yong Shin, Byoung-Kee Yi

**Affiliations:** 1 Samsung Advanced Institute for Health Sciences and Technology (SAIHST), Sungkyunkwan University, Seoul, 06355 Republic of Korea; 2 Department of Digital Health, SAIHST, Sungkyunkwan University, Seoul, 06355 Republic of Korea; 3 Research Institute for Future Medicine, Samsung Medical Center, Seoul, 06351 Republic of Korea; 4 Department of Artificial Intelligence Convergence, Kangwon National University, 1 Kangwondaehak-Gil, Chuncheon-si, Gangwon-do 24341 Republic of Korea

**Keywords:** Natural language processing, Deep learning, Data processing, Information extraction, Colonoscopy

## Abstract

**Background:**

Colorectal cancer is a leading cause of cancer deaths. Several screening tests, such as colonoscopy, can be used to find polyps or colorectal cancer. Colonoscopy reports are often written in unstructured narrative text. The information embedded in the reports can be used for various purposes, including colorectal cancer risk prediction, follow-up recommendation, and quality measurement. However, the availability and accessibility of unstructured text data are still insufficient despite the large amounts of accumulated data. We aimed to develop and apply deep learning-based natural language processing (NLP) methods to detect colonoscopic information.

**Methods:**

This study applied several deep learning-based NLP models to colonoscopy reports. Approximately 280,668 colonoscopy reports were extracted from the clinical data warehouse of Samsung Medical Center. For 5,000 reports, procedural information and colonoscopic findings were manually annotated with 17 labels. We compared the long short-term memory (LSTM) and BioBERT model to select the one with the best performance for colonoscopy reports, which was the bidirectional LSTM with conditional random fields. Then, we applied pre-trained word embedding using large unlabeled data (280,668 reports) to the selected model.

**Results:**

The NLP model with pre-trained word embedding performed better for most labels than the model with one-hot encoding. The F1 scores for colonoscopic findings were: 0.9564 for lesions, 0.9722 for locations, 0.9809 for shapes, 0.9720 for colors, 0.9862 for sizes, and 0.9717 for numbers.

**Conclusions:**

This study applied deep learning-based clinical NLP models to extract meaningful information from colonoscopy reports. The method in this study achieved promising results that demonstrate it can be applied to various practical purposes.

## Background

Colorectal cancer is a leading cause of cancer deaths [1–3]. Cancer screening helps in early cancer detection before the appearance of symptoms and reduces cancer mortality. Several screening tests, such as fecal occult blood test, fecal immunochemical test, and colonoscopy, can be used to find polyps or colorectal cancer. The US Preventive Services Task Force recommends colorectal cancer screening in adults aged 50 to 75. [4]. In the United States, colonoscopy prevalence among adults aged 50 years and above tripled from 20% in 2000 to 61% in 2018, primarily due to the Medicare expansion of colonoscopy screening coverage from high-risk individuals to all beneficiaries in 2011 [3]. Since 1999, the National Cancer Screening Program (NCSP) has been implemented in Korea, which provides free screening services for the six most common cancers: stomach, breast, colorectal, cervix, lung, and liver cancer [5]. According to the NCSP protocol, adults aged 50 years and above are eligible to take colorectal screening tests. The participation rates for colonoscopies increased from 25.0% in 2005 to 64.4% in 2020 [6]. The prevalence of colonoscopy, the most reliable way to prevent and detect colorectal cancer, has been increasing.

With the rapid adoption of electronic health records (EHRs), hospitals have accumulated a large amount of unstructured text data in EHR systems, such as discharge summaries, radiology reports, operation notes, and pathology reports. The unstructured text data in EHR systems contain clinically significant information, which is vital for comprehensive care. Regarding colonoscopies, the information embedded in the report can be used for various purposes, including colorectal cancer risk prediction, follow-up recommendations, and quality measurement [7–13]. The information embedded in colonoscopy reports can be used for various purposes, including colorectal cancer risk prediction, follow-up recommendations, and quality measurement. The location, size, number, and appearance of target lesions such as polyps, ulcers, stenosis, and bleeding can determine the risk of colorectal cancer and the follow-up treatment. The colonoscopic findings and procedural information can be used for the assessment of quality indicators, such as adenoma detection rate [14]. However, the availability and accessibility of the unstructured data are still insufficient despite the large amounts of accumulated data.

Natural language processing (NLP) is a computer science subfield that uses computational techniques to learn, understand, and produce human language content [15]. With the impressive advances of deep learning in computer vision and pattern recognition, the recent research in NLP is increasingly emphasizing the use of deep learning methods to overcome the drawbacks of traditional NLP systems, which depend heavily on the time-consuming and often incomplete hand-crafted features [16]. Although clinical NLP research has been actively performed since the 1960s, its progress was slow and lagged behind the progress of NLP in the general domain [17]. Similar to other areas, deep learning-based NLP research in the medical field has repeatedly demonstrated its feasibility [18–20].

Research data integration is essential in cancer research, and there are many efforts to gather and utilize clinical data, such as OHDSI CDM [21]. Although the importance of data has been increasing, many portions of EHR remain unstructured. Clinical NLP is the key to unlocking the evidence buried in clinical narratives. Unfortunately, clinical NLP research still faces several challenges, such as insufficient datasets or the complexity of clinical narratives [22–24]. Although certain pioneering efforts have made clinical text data available for sharing, the number of training datasets are relatively small for practical application. The representative clinical text datasets are MIMIC-III [25] and NLP community challenges, such as n2c2 NLP Research Data Sets [26], ShARe/CLEF eHealth [27], and CEGS N-GRID [19]. Besides, most of the shared datasets emphasize a single type of clinical narrative, like discharge summary [19], which does not reflect the characteristics of various medical specialties, for example, the different types of anatomical structures and their pathologies.

### Prior work

Clinical NLP research has recently emphasized the use of deep learning methods, and the publications are increasing yearly. Among deep learning models, recurrent neural network (RNN) has been widely employed in clinical NLP studies [18]. RNN [28] retains the memory of previous computations and uses it in current processing. Using this memory, RNN can capture the inherent sequential nature of language; therefore, it is suited for various NLP tasks such as named entity recognition (NER), machine translation, and speech recognition [16]. However, RNN suffers from the problem of vanishing and exploding gradients, which makes it challenging to learn and tune the parameters of the earlier layers in the networks. Its variants, such as long short-term memory (LSTM) [29] and gated recurrent unit (GRU) [30], have been proposed to overcome the limitation of RNN.

Clinical NER is an essential NLP task for extracting meaningful information from clinical narratives. Recently, numerous efforts have been made to combine RNN variants with other techniques, such as embedding techniques [31], attention mechanisms, and statistical modeling methods like CRFs [32, 33]. Among these techniques, word embedding (or distributed representation), such as Word2Vec [34], GloVe [35], and BERT [36], is a set of language modeling and feature learning techniques in NLP where words or phrases are mapped to a continuous vector space. Typically, word embedding is trained by optimizing an auxiliary objective in large unlabeled and semantic information [16]. Word embedding models trained by Word2Vec and GloVe assign the word to a certain vector, which means these models can only have context-independent representations [37]. BERT is one of the current state-of-the-art language models. Unlike traditional word embeddings such as Word2Vec and GloVe, BERT assign the word to embedding depending on the context, which means the word could have different representations in different contexts by utilizing a transformer network.

In previous studies, clinical standard terminologies such as UMLS or SNOMED CT have enriched word embedding using the semantic relations between clinical concepts [38, 39]. Although the embedding method using clinical standard terminologies is somewhat effective, it is unsuitable for dealing with various synonyms and abbreviated terms in colonoscopy reports. There are multiple expressions to describe colonoscopic findings; for example, "D-colon", "D colon", "Desc. colon", "DC", "mid-d-colon", and "proximal-d-colon" for descending colon; "T-ileum", "T ileum", "TI", and "T.I." for terminal ileum; and "H-flexure", "H flexure", "H-Fx", and "HF" for hepatic flexure. As mentioned in the result section, adding the pre-trained contextual information from the large unlabeled data to the embedding layer demonstrates a slightly better performance than merely learning with annotated data.

Several studies have been published on clinical NLP for colonoscopy, as shown in Table 1. The list of previous studies on clinical NLP for colonoscopies has been excerpted from Table 4 of Fevrier et al. [40], modified, and summarized in Table 1. Most studies have used statistical or rule-based NLP methods. Since there are no publicly available colonoscopy text data, all studies used data from each institution. Most of them focused on extracting information about polyps, such as presence, size, number, and type. Our study covered comprehensive endoscopic findings, such as stenosis, erosion, edema, ulcer, erythema, hyperemia, hemorrhage, and polyp. It isn't easy to directly compare the performance between this study and previous studies due to the difference in data sources and sizes.

**Table 1:** Previous studies on clinical NLP for colonoscopy reports

Year	Author	NLP method (tool)	NLP category
	Setting	Dataset	Performance
Current study	Seong et al	Bi-LSTM-CRF, BioBERT	Deep learning-based NLP
	Samsung Medical Center	280,668 colonoscopy reports Training and Test: 1,000–5,000 Embedding: 280,668	F1 score: 0.9564–0.9862
2022	Bae et al.[13]	SmartTA	Rule-based NLP (Commercial software)
	Seoul National University Hospital	54,562 colonoscopy reports and pathology reports Training: 2,000 Test: 1,000	Accuracy: 0.99–1.0
2021	Vadyala et al. [41]	Bio-Bi-LSTM-CRF	Deep learning-based NLP
	Veterans Affair Medical Centers (VA)	4,000 colonoscopy reports and pathology reports Training: 3,200 Test: 400 Validation: 400	F1 score: 0.85–0.964
2020	Fevrier et al. [40]	SAS PERL regular expression	Rule-based NLP (Commercial software)
	Kaiser Permanente Northern California (KPNC)	401,566 colonoscopy reports and pathology reports Training: 1,000 Validation: 3,000 Test: 397,566	Cohen's κ: 0.93–0.99
2020	Karwa et al. [12]	Prolog	Rule-based NLP (Logic program language)
	Cleveland Clinic	2,439 colonoscopy reports Validation: 263	Accuracy: 1.0
2019	Lee et al. [11]	Linguamatics I2E [42]	Rule-based NLP (Commercial software)
	Kaiser Permanente Northern California (KPNC)	500 colonoscopy reports Validation: 300	Accuracy: 0.893–1.0
2017	Hong et al. [10]	SAS ECC [43]	Rule-based NLP (Commercial software)
	Samsung Medical Center (SMC)	49,450 colonoscopy reports and pathology reports	Precision: 0.9927 Recall: 0.9983
2017	Carrell et al. [44]	HITEX [45]	Statistical NLP (Clinical NLP framework)
	University of Pittsburgh Medical Center (UPMC)	3,178 colonoscopy reports and 1,799 pathology reports Training: 1,051 Validation: 2,127	F-measure: 0.57–0.99
2015	Raju et al. [46]	CAADRR	Rule-based NLP
	MD Anderson	12,748 colonoscopy reports and pathology reports Validation: 343	Positive predictive value: 0.913
2014	Gawron et al. [47]	UIMA [48]	Statistical NLP (NLP framework)
	Northwestern University	34,998 colonoscopy reports and 10,186 pathology reports Validation: 200	F1 score: 0.81–0.95
2013–2015	Imler et al. [8, 9, 49]	cTAKES [50]	Statistical NLP (Clinical NLP framework)
	Veterans Administration medical center	42,569 colonoscopy reports and pathology reports Training: 250 Test: 500	Accuracy: 0.87–0.998
2011	Harkema et al. [51]	GATE [52]	Statistical NLP (NLP framework)
	University of Pittsburgh Medical Center (UPMC)	453 colonoscopy reports and 226 pathology reports	Accuracy: 0.89 (0.62–1.0) F-measure: 0.74 (0.49–0.89) Cohen’s κ: 0.62 (0.09–0.86)

### Objective

This study aimed to extract meaningful information from colonoscopy reports using deep learning approach. We applied pre-trained word embedding to a deep learning-based NER model using large unlabeled colonoscopy reports. We compared variants of the long short-term memory (LSTM) and BioBERT [53] model to select the one with the best performance for colonoscopy reports, which was the bidirectional LSTM with conditional random fields (CRF). Then we applied pre-trained word embedding using large unlabeled data to the selected model.

## Methods

### Data collection and text annotation

This study used colonoscopies performed at Samsung Medical Center from 2000 to 2015. Data for this study were extracted from DARWIN-C, the clinical data warehouse of Samsung Medical Center, launched in 2016. As shown in Table 2, the total number of extracted colonoscopy reports was 280,668, of which 5,000 reports from 2011 to 2015 were manually annotated using an open-source web-based text annotation tool named DOCCANO [54]. It provides annotation features for text classification, sequence labeling, and sequence-to-sequence tasks. We made the annotation based on the results of the DARWIN-C project. In the project, we performed text analysis to extract meaningful information from various clinical documents such as pathology, colonoscopy, gastro endoscopy, and radiology reports using a rule-based commercial software named SAS Enterprise Contents Categorization. The extracted results through the text analysis were reviewed and evaluated by clinicians of each department. In this study, two annotators performed the annotation using the tool DOCCANO, and then we manually reviewed all the annotations based on the results of the DARWIN-C project. All colonoscopy reports (280,668) were used for the pre-trained word annotation tool. All colonoscopy reports (280,668) were used for pre-trained word embedding, and the annotated reports (5,000) were used for training the NER model. Table 3 shows the statistics of data used for pre-trained word embedding and training and test.

**Table 2:** The number of extracted colonoscopy reports and annotated reports by year

Year	Colonoscopy reports	Annotated reports
2000	2,620	–
2001	3,521	–
2002	4,196	–
2003	4,890	–
2004	5,299	–
2005	7,780	–
2006	9,525	–
2007	10,926	–
2008	17,108	–
2009	26,617	–
2010	30,387	–
2011	34,446	1,000
2012	32,441	1,000
2013	32,103	1,000
2014	34,156	1,000
2015	24,653	1,000
Total	280,668	5,000

**Table 3:** Data statistics

Data	For pre-trained word embedding	For training and test
Year	2000–2015	2011–2015
Number of documents	280,668	5,000
Number of sentences	4,193,814	81,666
Number of types of words	41,563	4,478

In general, a colonoscopy report includes various information, such as patient information (indication/reason), procedural information, colonoscopic findings, and assessment (interpretation, conclusion, and recommendation) [55, 56]. Among the several items that describe the result of colonoscopy, the items for colonoscopic findings are an essential part of the colonoscopy report. Table 4 lists the generally used items in a colonoscopy report; labels were assigned to the items to be extracted. A total of 17 labels were used in this study. As shown in Table 4, our study covered comprehensive endoscopic findings. For the lesion of the colonoscopic findings in Table 4, there are two labels; Lesion and Negation. The label "Lesion" presents the presence of lesions and abnormalities. The negation scope of this study is the absence of any lesions, abnormalities, or tumor recurrence. Finding the absence of lesions or tumor recurrence is crucial for determining cancer diagnosis. There are several patterns for negation clues to describe the absence in colonoscopy reports [57]. For example, "There was no evidence of tumor recurrence.", "There was no mucosal lesion.", "There was no other mucosal abnormality." and "There was no definite mass lesion.". But this study excluded a few items like family history, indication, and withdrawal time because most of our colonoscopy reports did not fully describe the information.

**Table 4:** Items of the colonoscopy report and assigned labels for annotation

Items	Labels ^a^
*1. Patient information*	
1.1 Brief history (disease, family, etc.)	
1.2 Indication/reason for endoscopy	
*2. Procedures*	
2.1 Sedation and other drugs	
2.1.1 Sedation	SEDATION
2.1.1.1 Level of sedation	SEDATIONLEVEL
2.1.1.2 Medication	MEDICATION
2.1.1.3 Dosage	DOSAGE
2.1.2 Antispasmodics	ANTISPASMODICS
2.2 Equipment (endoscope) used	DEVICE
2.2.1 Extent of examination	EXTENT
2.3 Quality of cleansing/visualization	PREPARATION
2.4 Procedural time	
2.4.1 Time-to-cecum	
2.4.2 Withdrawal time	
2.5 Digital rectal examination	DRE
*3. Colonoscopic findings*	
3.1 Lesions and their attributes	
3.1.1 Lesion	LESION, NEGATION ^b^
3.1.2 Anatomical site	LOCATION
3.1.3 Shape	SHAPE
3.1.4 Color	COLOR
3.1.5 Size	SIZE
3.1.6 Number	NUMBER
3.2 Sampling (type of sample)	BIOPSY
3.3 Adverse intraprocedural events	
*4. Conclusion*	

^a^A total of 17 labels are used in this study

^b^"NEGATION" is used to detect negated concepts

As shown in Textbox 1, the colonoscopy report can be divided into two parts: procedures and colonoscopic findings. Procedural information is written in semi-structured text (e.g., Level of sedation: Moderate). On the other hand, colonoscopic findings are often written in free text (e.g., On the distal descending colon, about 0.5 cm sized Is polyp was noticed.). Colonoscopic findings include lesions and their attributes to describe the lesions, such as their anatomical site, size, number, shape, and color. Colonoscopic findings are more complex than procedures, and the terms used for colonoscopic findings are often written using various expressions. For example, one of the anatomical sites, the ascending colon, is written in an unexpected form such as "a-colon", "a colon", "a. colon", "ac", "a. c.", and "a-c". As will be shown later, the accuracy of the extraction of procedural information was much better than that of colonoscopic findings. Our corpus contains a few discontinuous entities, though not many. We assigned the labels two ways for the discontinuous entities: grouping them together or separating them. For example, "on the ascending colon and descending colon" is divided into two entities: "ascending colon" and "descending colon". On the other hand, "on the ascending and descending colon" is assigned to an entity: "ascending and descending colon".

**Textbox 1:** An example of the colonoscopy report with annotations. Italicized text indicates the target of annotation, and the terms in square brackets are labels for annotation

*Clinical information* Past (medical) Hx: AGC s/p STG B-II Antithrombotics: No Indication: Checkup *Procedure Note* Sedation: *Yes* [SEDATION]: *midazolam* [MEDICATION] *3 mg* [DOSAGE] *pethidine* [MEDICATION] *50 mg* [DOSAGE] Level of sedation: *moderate* [SEDATIONLEVEL] (paradoxical response: no) Antispasmodics (cimetropium 5 mg): *Yes* [ANTISPASMODICS] Digital rectal examination was *normal* [DRE] Bowel preparation was *fair* [PREPARATION] The *CF 260AI* [DEVICE] was inserted up to the *terminal ileum* [EXTENT] *Colonoscopic finding* On the *terminal ileum* [LOCATION], *several* [NUMBER] *erosions* [LESION] and *shallow* [SHAPE] *ulcer* [LESION] were noticed There were *several* [NUMBER] *outpouching lesions* [LESION] on the *ascending colon* [LOCATION]. On the *distal descending colon* [LOCATION], about *0.5 cm* [SIZE] sized *Is* [SHAPE] *polyp* [LESION] was noticed. It was removed by cold biopsy. On the *rectum* [LOCATION], *AV 10 cm* [LOCATION] about *0.3 cm* [SIZE] sized *Is* [SHAPE] *polyp* [LESION] was noticed. It was removed by cold biopsy biopsy + [BIOPSY] *Conclusion* 1. Colon polyp, removed 2. Rectal polyp, removed 3. A-colon diverticulum *Comment* No immediate complication	

An open-source web-based text annotation tool was used to create training and test datasets. As shown in Table 5, we made five different sizes of annotated datasets which were increased by 1,000, to compare the performance according to the amount of data. The datasets D1, D2, D3, and D4 were randomly generated from 5,000 annotated data. Table 6 shows the distribution of the assigned labels of the datasets. Most of the experiments were performed with dataset D1, except for the performance comparison according to the amount of data. We applied fivefold cross-validation to evaluate the model using all parts of the data. The output file of the text annotation tool was JSON formatted file. It was converted to IOB2 formatted data, where B refers to the beginning of the phrase, I the elements within the phrase, and O the elements outside the phrase [58]. Each token is classified using an IOB label. For example, "on the ascending colon" with the "LOCATION" label was tagged as "O O B-LOC I-LOC". We used partial matches to calculate the performance for the entity consisting of several tokens.

**Table 5:** Training and test datasets

Dataset ^a^	D1	D2	D3	D4	D5
Number of documents	1,000	2,000	3,000	4,000	5,000
Number of sentences	16,417	32,821	49,048	65,279	81,668
Number of words	92,315	184,928	277,266	369,063	461,713
Number of types of words	2,001	2,771	3,410	3,922	4,478

^a^The dataset sizes were increased by 1,000 to compare the performance according to the amount of data. For evaluation, fivefold cross-validation was applied

**Table 6:** Training and test datasets

Labels	D1	D2	D3	D4	D5
*PROCEDURE NOTE*					
SEDATION	860	1,735	2,586	3,443	4,312
SEDATIONLEVEL	679	1,361	2,027	2,706	3,404
MEDICATION	871	1,778	2,659	3,566	4,500
DOSAGE	872	1,781	2,663	3,576	4,515
ANTISPASMODICS	799	1,620	2,408	3,215	4,032
DRE	995	1,990	2,986	3,982	4,979
PREPARATION	996	1,993	2,985	3,977	4,971
DEVICE	999	2,000	2,997	3,994	4,992
EXTENT	1,000	2,000	2,998	3,995	4,992
*COLONOSCOPIC FINDINGS*					
LESION	1,043	2,053	3,201	4,237	5,336
LOCATION	1,118	2,269	3,481	4,599	5,757
SHAPE	719	1,513	2,296	3,024	3,795
COLOR	197	373	589	789	983
SIZE	726	1,530	2,318	3,037	3,831
NUMBER	219	416	639	853	1,052
BIOPSY	995	1,993	2,991	3,987	4,984
NEGATION	651	1,300	1,929	2,609	3,240
Total	13,739	27,705	41,753	55,589	69,675

### Model

**Fig. 1:**
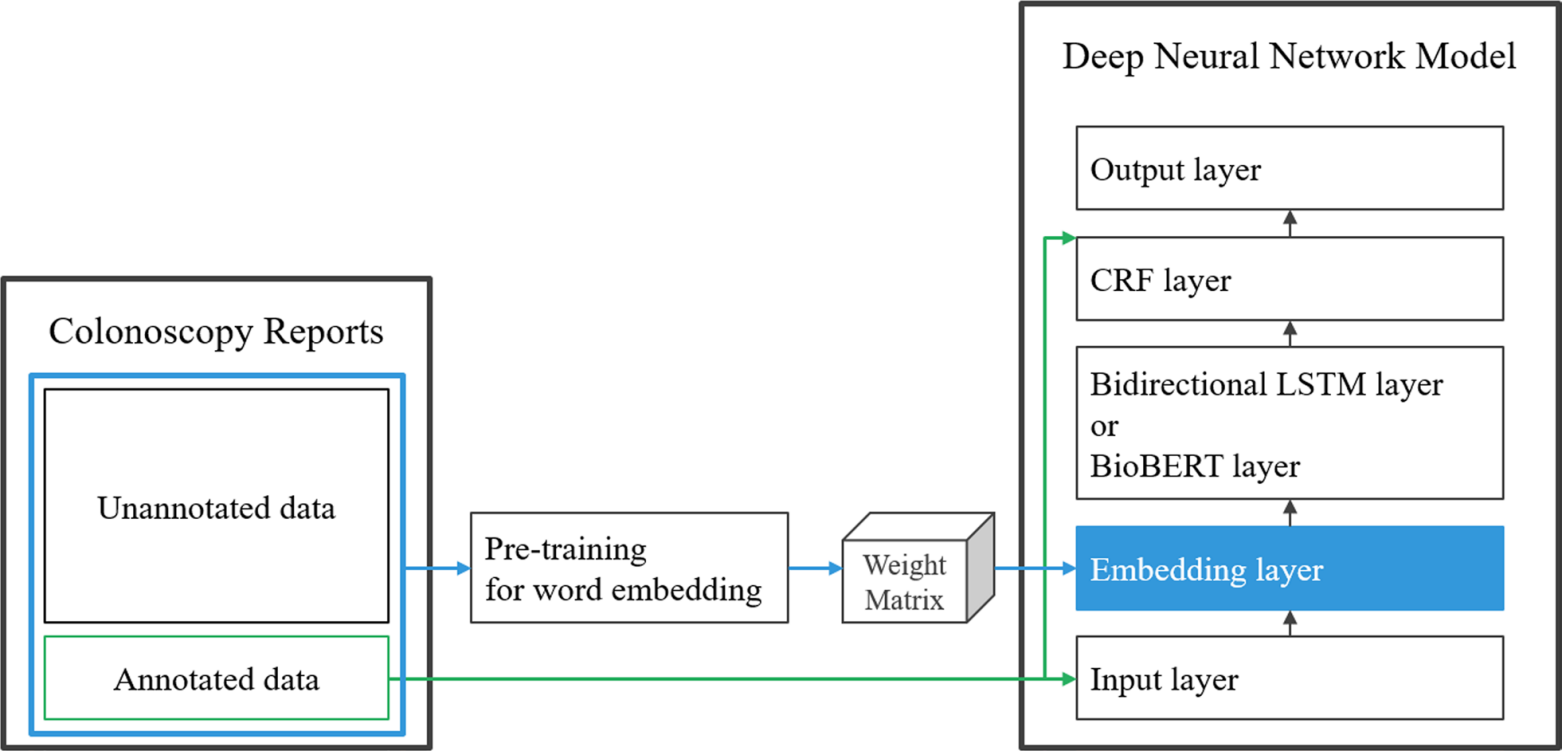
The architecture of bidirectional LSTM-CRF and BioBERT with pre-trained word embedding using unannotated data

Figure 1 presents the overall architecture of the model. We selected the Bi-LSTM with a CRF layer (Bi-LSTM-CRF) and BioBERT as the model for the current study. It largely contained three layers: input and embedding layer, bidirectional LSTM layer or BioBERT layer, and CRF layer. Annotated data was composed of a set of words and labels used as input and output of the model. A pre-trained word embedding using unannotated data was applied to the embedding layer.

#### Input and embedding layer

To prepare a sequence of tokens for the input layer, documents were split into sentences, and sentences were zero-padded to make the input equal in length. Special characters were removed within the criteria that do not alter the number and position of the words and sentences. For the input representation, we assigned each word with an integer value and then converted the unique integer value to a binary vector.

The weight matrix of the input word representation was created using Word2Vec [59], which uses a neural network model to learn word association from a large unlabeled corpus of text. Word2Vec utilizes continuous bag-of-words (CBOW) and skip-gram models. We applied the CBOW model, which learns the conditional probability of a target word given the context words surrounding it across a window. As shown in Table 3, 41,563 words (280,668 colonoscopy reports) were used for training the weight matrix. The embedding layer was seeded with the weight matrix, and the input words were mapped to word vectors.

#### Bidirectional LSTM layer

NER is a task for identifying meaningful words or phrases in a given text and classifying them into predefined semantic categories. Therefore, we focused on the principle of LSTM [29] to capture the context of the sentence and extract the meaning of each word from the sentence. LSTM is a variant of RNN composed of a cell, and three gates: input, forget, and output. The cell captures the long-term dependencies over any time interval, and the three gates regulate the flow of information into and out of the cell. This unique mechanism can effectively memorize the context of the entire input sentence and overcome vanilla RNN's vanishing and exploding gradient problem. Based on this principle, we constructed a bidirectional LSTM (Bi-LSTM) [60] layer to jointly capture past and future features to obtain a better text representation.

#### BioBERT layer

BERT utilizes a transformer network to pre-train a language model by jointly conditioning on both the left and right context in all layers. The transformer model introduces a multi-layer, multi-head self-attention mechanism that has demonstrated superiority over RNNs and LSTMs in exploiting GPU-based parallel computation and modeling long-range dependencies in a text [61]. The original BERT model was trained from general domain knowledge, such as Wikipedia and BookCorpus. According to the need for models that can perform better for each domain, domain-specific models such as BioBERT and ClinicalBERT have been developed [53, 62]. This study used an existing pre-trained contextualized word embedding, BiomedNLP-PubMedBERT, which was pre-trained using abstracts from PubMed and full-text articles from PubMedCentral.

#### CRF layer

The output of the Bi-LSTM was used as input to the CRF layer. CRFs [63], one of the often used statistical modeling methods in the field of NLP [64], are used for structured prediction. CRFs learn the dependencies between labels (i.e., IOB constraints) from training data. For example, I-tag does not appear in the first word of the phrase, and the O-I pattern does not exist in the IOB format. As will be shown later, the model with a CRF layer performs much better than learning without the layer.

### Experiment

**Fig. 2:**
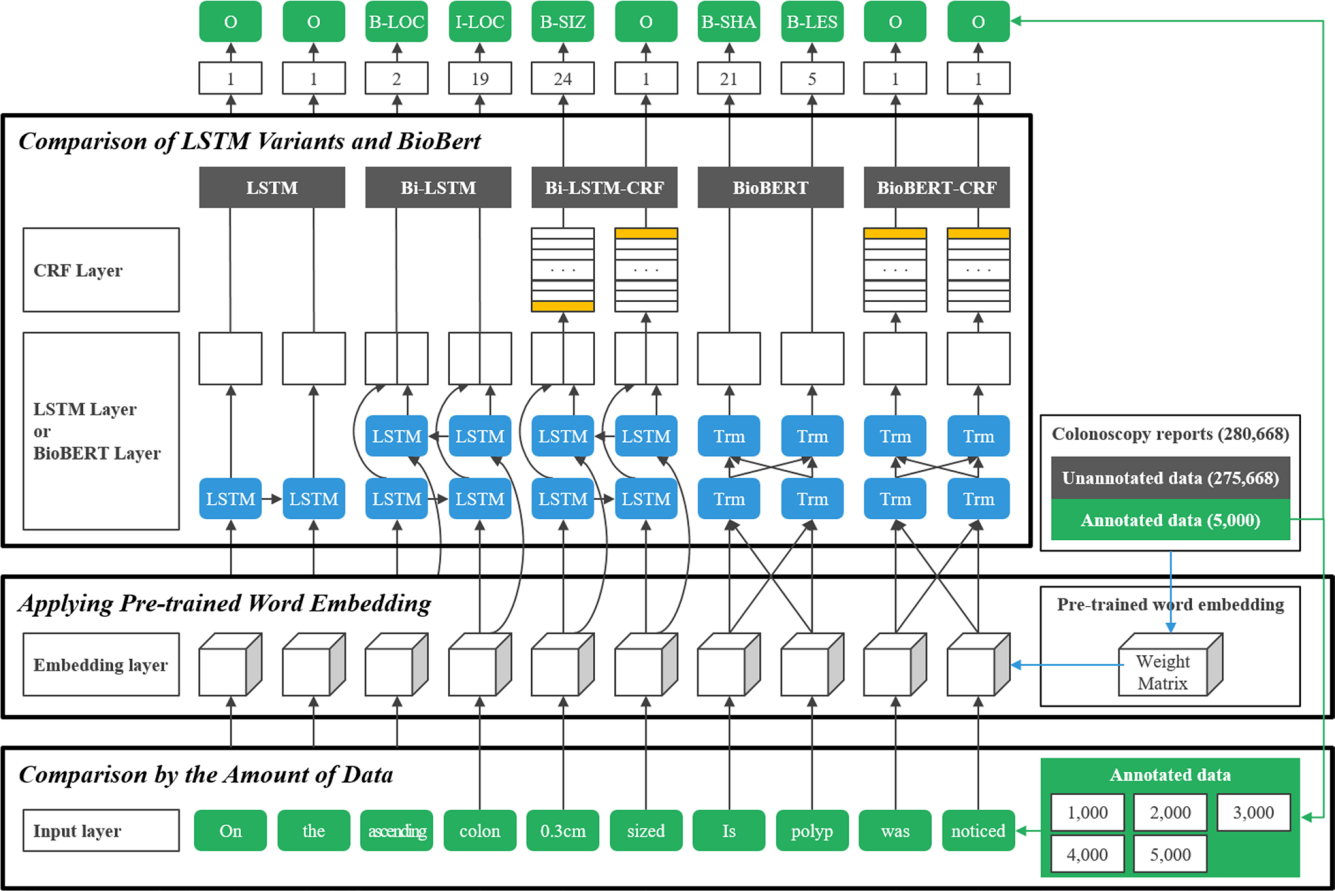
Three experiments performed in this study

This study conducted three experiments, as shown in Fig. 2. First, we compared variants of the LSTM and BioBERT model to the one with the best performance for colonoscopy reports. Then, we applied pre-trained word embedding using unannotated data to the selected model. Additionally, we compared the effect on performance as the training data increased.

#### Comparison of LSTM and BioBERT variants

We compared LSTM, Bi-LSTM, Bi-LSTM-CRF, BioBERT, and BioBERT-CRF models with different loss functions and optimizers to select the appropriate model and parameters. For the loss function, Categorical Cross-Entropy (CCE), Kullback–Leibler (KL) divergence [65], and Poisson distribution were used. The CRF layer uses its loss function to learn a set of transition probabilities. For the optimizer, Adaptive Moment Estimation (ADAM) [66], Nesterov-accelerated Adaptive Moment Estimation (NADAM) [67], and Root Mean Square Propagation (RMSProp) [68] were used. For the BioBERT model, we used an existing pre-trained contextualized word embedding, BiomedNLP-PubMedBERT, which was pre-trained using abstracts from PubMed and full-text articles from PubMedCentral [61, 69]. The dataset D1 presented in Table 5 was used in this experiment, and one-hot encoding was used for the input word representation. The experimental parameters were 128 as the dimension of embedding, 256 as the dimension of the LSTM hidden layer, and ten as the epoch.

#### Applying pre-trained word embedding

As shown in Table 3, about 280,668 colonoscopy reports were trained using Word2Vec to demonstrate the effect of pre-trained word embedding. The CBOW training algorithm was used. For comparison, one-hot encoding and pre-trained word embedding were applied to the Bi-LSTM-CRF model with RMSProp in Table 7, respectively. The dataset D1 presented in Table 5 was used in this experiment. The experimental parameters were 128 as the dimension of word embedding, five as the size of the window, and three as the minimum count of words.

**Table 7:** Comparison of LSTM and BioBERT variations

Model	Loss function ^a^ & optimizer ^b^	Precision ^c^	Recall ^c^	F1 score ^c^
LSTM	CCE + ADAM	0.5267	0.5297	0.5282
LSTM	CCE + NADAM	0.5258	0.5285	0.5271
LSTM	CCE + RMS	0.5266	0.5297	0.5281
LSTM	KL + ADAM	0.5255	0.5286	0.5270
LSTM	KL + NADAM	0.5258	0.5287	0.5273
LSTM	KL + RMS	0.5260	0.5278	0.5269
LSTM	POISSON + ADAM	0.5255	0.5274	0.5264
LSTM	POISSON + NADAM	0.5245	0.5267	0.5256
LSTM	POISSON + RMSProp	0.5229	0.5258	0.5244
Bi-LSTM	CCE + ADAM	0.5880	0.6761	0.6290
Bi-LSTM	CCE + NADAM	0.5971	0.7056	0.6460
Bi-LSTM	CCE + RMSProp	0.5884	0.6763	0.6293
Bi-LSTM	KL + ADAM	0.5881	0.6768	0.6294
Bi-LSTM	KL + NADAM	0.5957	0.7039	0.6445
Bi-LSTM	KL + RMSProp	0.5884	0.6767	0.6295
Bi-LSTM	POISSON + ADAM	0.5873	0.6756	0.6284
Bi-LSTM	POISSON + NADAM	0.5949	0.7021	0.6433
Bi-LSTM	POISSON + RMSProp	0.5869	0.6758	0.6282
Bi-LSTM-CRF	CRF + ADAM	0.9828	0.9842	0.9835
Bi-LSTM-CRF	CRF + NADAM	0.9825	0.9851	0.9838
Bi-LSTM-CRF	CRF + RMSProp	**0.9844**	**0.9853**	**0.9848**
BioBERT	CCE + ADAM	0.9824	0.9821	0.9822
BioBERT-CRF	CRF + ADAM	0.9810	0.9815	0.9812

^a^Loss functions; CCE = categorical cross-entropy, KL = Kullback–Leibler divergence, POISSON = Poisson distribution

^b^Optimizers; ADAM = Adaptive Moment Estimation, NADAM = Nesterov-accelerated Adaptive Moment Estimation, RMSProp = Root Mean Square Propagation

^c^The best results are marked in bold

#### Comparison by the amount of data

The datasets D1, D2, D3, D4, and D5 in Table 5 were used to compare the effect on performance as the amount of training data increased. The Bi-LSTM-CRF model with pre-trained word embedding in Table 8 was used in this experiment.

**Table 8:** Comparison between one-hot encoding and pre-trained word embedding

Labels	Bi-LSTM-CRF + one-hot encoding			Bi-LSTM-CRF + pre-trained word embedding		
	Precision	Recall	F1 score	Precision	Recall	F1 score
*PROCEDURE NOTE *^*a*^						
SEDATION	0.9881	0.9953	0.9916	0.9888	0.9950	**0.9918**
SEDATIONLEVEL	0.9987	0.9938	0.9962	0.9985	0.9958	**0.9971**
MEDICATION	0.9991	0.9954	0.9972	1	0.9959	**0.9980**
DOSAGE	0.9929	0.9897	0.9913	0.9959	0.9920	**0.9939**
ANTISPASMODICS	0.9962	1	0.9981	0.9978	1	**0.9989**
DRE	0.9967	0.9990	**0.9978**	0.9958	0.9989	0.9973
PREPARATION	0.9892	0.9914	0.9903	0.9879	0.9928	**0.9904**
DEVICE	0.9991	0.9991	**0.9991**	0.9980	0.9979	0.9979
EXTENT	0.9883	0.9951	0.9916	0.9960	0.9967	**0.9963**
*COLONOSCOPIC FINDINGS *^*b*^						
LESION	0.9881	0.9953	0.9916	0.9888	0.9950	**0.9918**
LOCATION	0.9987	0.9938	0.9962	0.9985	0.9958	**0.9971**
SHAPE	0.9991	0.9954	0.9972	1	0.9959	**0.9980**
COLOR	0.9929	0.9897	0.9913	0.9959	0.9920	**0.9939**
SIZE	0.9962	1	0.9981	0.9978	1	**0.9989**
NUMBER	0.9967	0.9990	0.9978	0.9958	0.9989	**0.9973**
BIOPSY	0.9892	0.9914	0.9903	0.9879	0.9928	**0.9904**
NEGATION	0.9991	0.9991	0.9991	0.9980	0.9979	**0.9979**
MICROAVG	0.9883	0.9951	0.9916	0.9960	0.9967	**0.9963**

^a^Procedure note was written in semi-structured text. The best results are marked in bold

^b^Colonoscopic findings were written in free text. The best results are marked in bold

## Results

### Comparison of LSTM and BioBERT variants

In Table 7, the experimental result shows the performance of LSTM variants with the combinations of loss functions and optimizers. As shown in Table 7, the Bi-LSTM-CRF model with RMSProp achieved the best performance with a precision of 0.9844, a recall of 0.9853, and an F1-score of 0.9848. The model with a CRF layer performed much better than the others. The CRF layer was a vital component in the NER problem. There was little difference in the performance depending on the loss functions and optimizers. We also compared GRU variants; there was no significant difference in the performance between LSTM and GRU models. All measures in Table 7 were micro-averaged to capture each label imbalance adequately.

### Applying word embedding

**Fig. 3:**
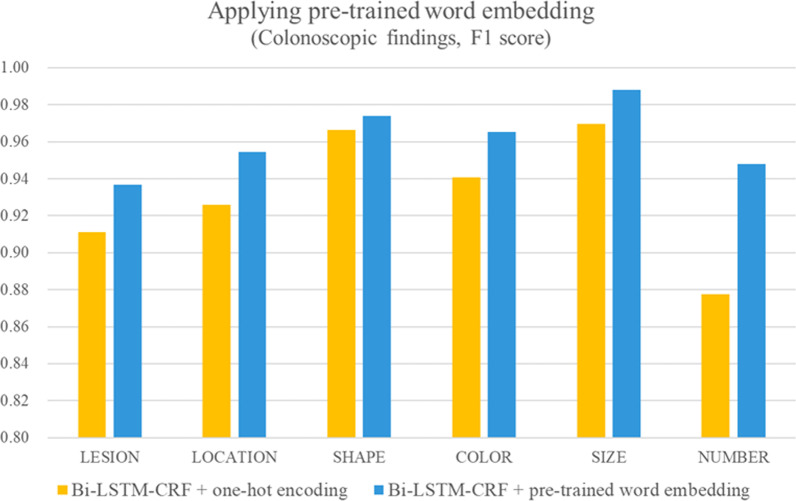
Performance of pre-trained word embedding

About 280,668 colonoscopy reports were trained using Word2Vec to demonstrate the effect of pre-trained word embedding. Table 8 shows the performance of each label; the model with pre-trained word embedding performed better for most labels than the model with one-hot encoding. In the case of the labels for procedure notes, both one-hot encoding and pre-trained word embedding had F1 scores of more than 0.99 because the procedure note was written in semi-structured text. In the case of the labels for colonoscopic findings, adding pre-trained word embedding improved the performance at a certain rate. Figure 3 shows the effect of pre-trained word embedding for colonoscopic findings.

### Comparison by the amount of data

**Fig. 4:**
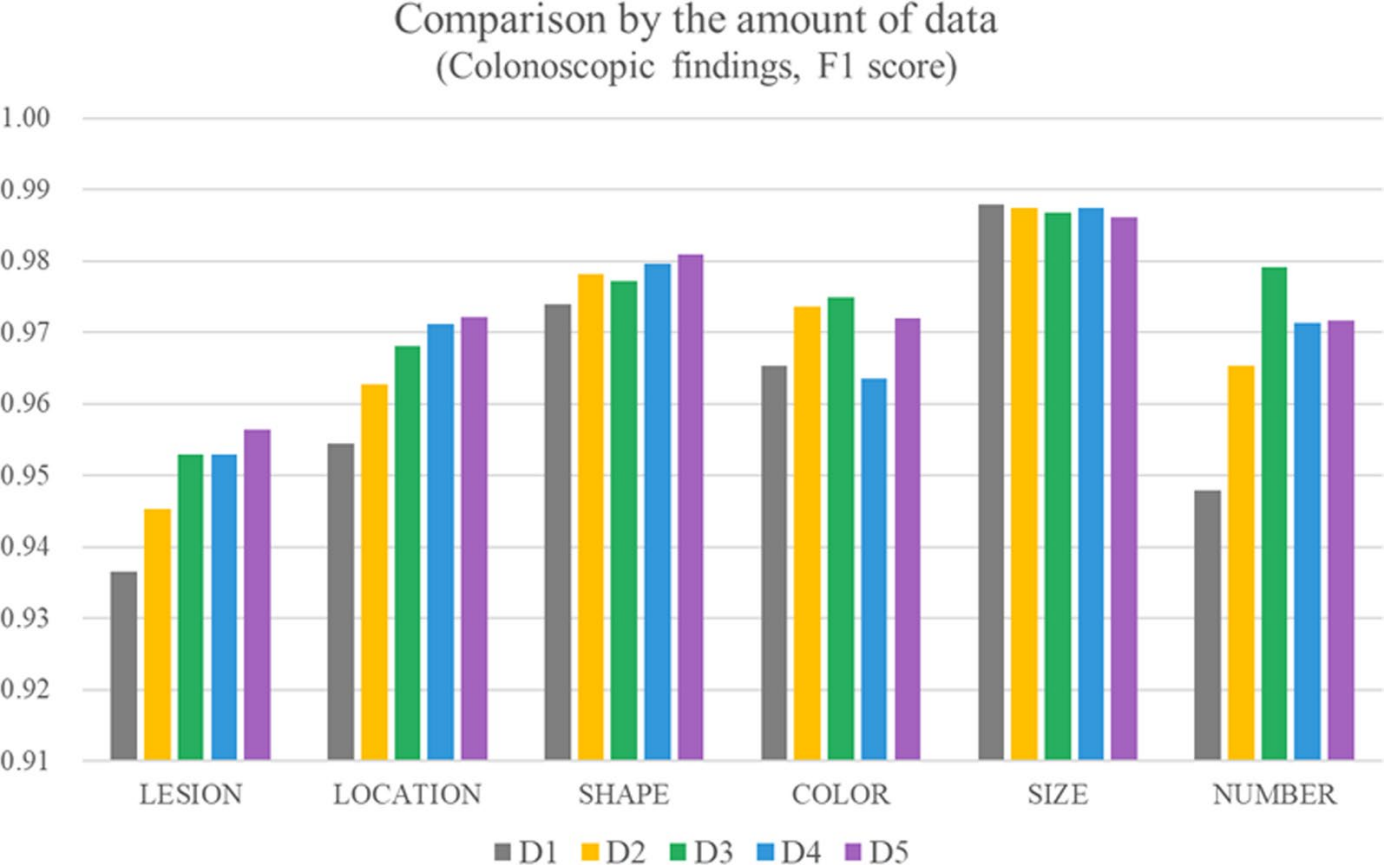
Comparison by the amount of data

As shown in Table 9, the performance slightly improved as data increased. As previously mentioned, F1 scores of the labels for procedure notes were more than 0.99 due to semi-structured patterns. As shown in Fig. 4, F1 scores of LESION, LOCATION, and SHAPE improved as the amount of data increased. COLOR and NUMBER had the best F1 scores in D3. SIZE had a similar performance for all data.

**Table 9:** Comparison by the amount of data (F1 score)

Labels	D1	D2	D3	D4	D5
*COLONOSCOPIC FINDINGS *^*a*^					
LESION	0.9366	0.9453	0.9530	0.9530	**0.9564**
LOCATION	0.9545	0.9627	0.9681	0.9711	**0.9722**
SHAPE	0.9739	0.9782	0.9772	0.9797	**0.9809**
COLOR	0.9653	0.9736	**0.9749**	0.9636	0.9720
SIZE	**0.9879**	0.9874	0.9867	0.9875	0.9862
NUMBER	0.9480	0.9653	**0.9791**	0.9713	0.9717
BIOPSY	0.9975	0.9985	0.9988	0.9989	**0.9992**
NEGATION	0.9772	0.9784	0.9845	0.9815	**0.9858**
MICROAVG	0.9892	0.9912	0.9921	0.9921	**0.9924**

^a^The best results are marked in bold

As an analysis of errors of this model, the errors came from various reasons, such as vocabulary: synonyms, acronyms, and typos (i.e., easy touch bleeingd), grammatical mistakes (i.e., from cecum ~ t colon), and incorrect extraction of size and location (i.e., on the 80 cm from anal verge).

## Discussion

This study has shown the feasibility of deep learning-based NLP methods to extract meaningful information from colonoscopy reports. Since there are no publicly available colonoscopy text data, 5,000 out of 280,668 colonoscopy reports were manually annotated using an open-source web-based text annotation tool. In the initial planning phase of this study, we assumed that 275,668 unannotated data could improve performance because it contained real-world synonyms and acronyms used in our institution. We compared LSTM, Bi-LSTM, Bi-LSTM-CRF, BioBERT, and BioBERT-CRF models to select the one with the best performance for colonoscopy reports. Although BioBERT performed much better than Bi-LSTM without a CRF layer, the performance of Bi-LSTM with a CRF layer was slightly higher than others. We applied pre-trained word embedding using large unlabeled data to the Bi-LSTM-CRF model. Therefore, the NER model with pre-trained word embedding performed better for most labels than the model without pre-trained word embedding. Although the deep learning-based NLP method performs much better than the traditional NLP method, the currently available public data is insufficient to cover the characteristics of various medical specialties. The method in this study could be effective in the absence of a shared colonoscopy dataset.

This study has the following limitations. First, the study was conducted in a single institution, so it is possible that the model could not handle colonoscopy reports from other institutions. They can differ in many aspects, such as writing patterns, templates, and vocabulary. Although our model may not apply to other colonoscopy reports directly, our approach can be used in others. Second, there are no available colonoscopy datasets to compare the performance of our model. Evaluating the performance of the model is not possible, but we believe that the performance level of our model is sufficient for clinical applications. Third, we need to consider synonyms, acronyms, and typos and be able to process them. There are various synonyms and acronyms to describe colonoscopic findings and anatomical sites; for example, "D-colon", "D colon", "Desc. colon", "DC", "mid-d-colon", and "proximal-d-colon" for descending colon; "T-ileum", "T ileum", "TI", and "T.I." for terminal ileum; and "H-flexure", "H flexure", "H-Fx", and "HF" for hepatic flexure.

## Conclusions

Realizing the full potential of precision medicine begins with identifying better ways to collect, share, and make decisions based on data [70]. Although the importance of data has been increasing, clinical data is still challenging to use as many portions of EHR remain unstructured. Clinical NLP is the key to unlocking the evidence buried in clinical narratives. With the advancements in deep learning techniques, NLP research has achieved remarkable performance in the clinical domain. However, obtaining sufficient public data for training is difficult. The currently available public data is insufficient to cover the characteristics of various medical specialties.

This study addresses the first deep learning-based NLP method for NER in colonoscopy reports. It is an important problem for clinical utility, such as cancer risk prediction, follow-up recommendation, and quality measurement. We applied a deep learning-based clinical NER with pre-trained word embedding. The method in this study achieves promising results that demonstrate it can be helpful for various practical purposes, including clinical document summarization and automated structured report generation.

## Data Availability

The datasets generated and analyzed during the current study are not publicly available because of privacy issues but are available from the corresponding author upon reasonable request. The code implementation of this study is publicly accessible from GitHub, https://github.com/dhseong/Deep-Learning-based-NLP-for-Colonoscopy [71].

## Abbreviations

ADAM: Adaptive moment estimation
Bi-LSTM: Bidirectional LSTM
Bi-LSTM-CRF: Bidirectional LSTM with CRF layer
CBOW: Continuous bag-of-words
CCE: Categorical cross-entropy
CRF: Conditional random fields
EHR: Electronic health record
GRU: Gated recurrent unit
KL: Kullback–Leibler divergence
LSTM: Long short-term memory
NADAM: Nesterov-accelerated adaptive moment estimation
NCSP: National cancer screening program
NER: Named entity recognition
NLP: Natural language processing
RMSProp: Root mean square propagation
RNN: Recurrent neural network
